# Evaluation of the Receptor Activator of Nuclear Factor Kappa B Ligand (*RANKL*) Expression in Osteosarcoma and Its Association with the Clinicopathological Data

**DOI:** 10.31557/APJCP.2021.22.3.741

**Published:** 2021-03

**Authors:** Hui Heng Chua, Sharifah Emilia Tuan Sharif, Wan Faisham Nu’man Wan Ismail, Muhamad Syahrul Fitri Zawawi, Sarimah Abdullah

**Affiliations:** 1 *Department of Pathology, School of Medical Sciences, Universiti Sains Malaysia, 16150 Kubang Kerian, Kelantan, Malaysia. *; 2 *Hospital Universiti Sains Malaysia, Health Campus, Universiti Sains Malaysia, 16150 Kubang Kerian, Kelantan, Malaysia. *; 3 *Department of Orthopaedic, School of Medical Sciences, Universiti Sains Malaysia, 16150 Kubang Kerian, Kelantan, Malaysia. *; 4 *Department of Biostatistics and Research Methodology Unit, School of Medical Sciences, Universiti Sains Malaysia, 16150 Kubang Kerian, Kelantan, Malaysia. *

**Keywords:** RANKL, immunohistochemistry, sarcoma, prognosis

## Abstract

**Background:**

The receptor activator of nuclear factor kappa B ligand (RANKL) is one of the key regulators of bone remodelling in bone oncology, including osteosarcoma. We assessed RANKL immunohistochemical expression in osteosarcoma, its association, and disease-free survival with the patients’ clinicopathological characteristics.

**Methods:**

One hundred osteosarcoma cases from 2003 to 2018 in Hospital Universiti Sains Malaysia were retrieved. The tissue samples were stained for RANKL, and the association with the clinicopathological characteristics was evaluated. Staining was interpreted in a semiquantitative scoring system and classified into negative and positive expressions.

**Results:**

Eighty-two cases had a positive expression of *RANKL* in which 56 and 26 patients were classified as low expression and high expression, respectively. The positive expression of RANKL did not show a significant association with clinicopathological characteristics. However, Kaplan Meier survival analysis showed a significant improvement in the disease-free survival patients who underwent limb salvage surgery (LSS) than amputated patients (p-value 0.002), whereas poorer survival was observed among conventional osteosarcomas compared to non-conventional osteosarcoma (p-value 0.01).

**Conclusion:**

Limb salvage surgery had proven to improve osteosarcoma patients’ survival compared to amputation, which could improve overall quality of life in osteosarcoma patients. However, our data did not show a significant association between positive RANKL immunohistochemistry with any of the clinicopathological characteristics and patients’ final survival. Further studies may be acquired to understand the suitability of using RANKL immunohistochemistry as prognostication in the management of osteosarcoma patients.

## Introduction

Osteosarcoma is a rare sarcoma that has histological findings of osteoid production in association with malignant mesenchymal cells (Misaghi et al., 2018). The incidence of osteosarcoma in all populations worldwide is approximately 4-5 per 1,000,000 population (Fletcher et al., 2002). Osteosarcoma represents the most common primary bone malignancy (Savage and Mirabello, 2011), wherein Malaysia, it accounts for 52.5% of all bone cancer cases from 2007 to 2011 (Azizah et al., 2016). When a wide range of ages are combined, males are affected more frequently than females (Savage and Mirabello, 2011). The typical presentation includes the onset of pain and swelling in the affected bone, occasionally associated with a pathologic fracture (Isakoff et al., 2015). Osteosarcoma occurs most frequently in the distal femur (43%), proximal tibia (23%), and proximal humerus (10%). Approximately 15–20% of patients have metastases detectable at presentation, with the lung being the most common site of distant disease (85%) (Gill et al., 2013). 

A meta-analysis reported significant survival gains were made for patients with osteosarcoma from the 1960s to the 1980s due to the introduction of multi-agent chemotherapy and gradually improved surgical techniques. Recurrence rates decreased in the 1970s, and then further outcome improvements appeared to have plateaued. The plateau was due to the main chemotherapeutic agents used to treat osteosarcoma had not changed much for the past 30 years (Allison et al., 2012). Therefore, the identification of novel targeted therapeutic approaches to improve the survival of the patients suffering from osteosarcoma is now needed to understand the biology of osteosarcoma is a prerequisite. 

The receptor activator of nuclear factor kappa B (RANK)- RANK ligand (RANKL) -osteoprotegerin (OPG) pathway represents a key regulator of bone metabolism, both in normal and pathological conditions (Santini et al., 2011). RANKL is a cytokine that plays an essential role in signalling the terminal differentiation of monocytes/macrophages into osteoclasts (Takayanagi et al., 2002). Thus, the resorption that occurs during both bone development and remodelling is orchestrated by matrix-embedded osteocytes and chondrocytes via the production of the RANKL for osteoclast differentiation and function (Xiong et al., 2011). RANKL plays a crucial role in cell migration and tissue-specific metastatic behaviour of cancer cells (Mori et al., 2007b). OPG, a secreted decoy receptor that negatively regulates RANKL, binds to RANK, thereby inhibiting bone resorption by osteoclasts (Mori et al., 2007a). Studies have reported the expression of *RANKL* in osteosarcoma, and the proportion ranges from 8.8% to 75% (Lee et al., 2011; Bago-Horvath et al., 2014; Branstetter et al., 2015). *RANKL* expression among osteosarcoma patients was found to be related to poor response to chemotherapy (Bago-Horvath et al., 2014) and had lower 5-year event-free survival (Lee et al., 2011). 

Denosumab, a humanized monoclonal antibody to RANKL, which inhibits bone resorption by osteoclasts, has shown its efficacy in clinical trials of giant cell tumour of bone (Heymann, 2012) and significantly reduced or eliminated RANK positive tumour giant cells (Branstetter et al., 2012). An investigation on the expression of *RANKL* in osteosarcoma might give similar benefits to improve the prognosis of patient with osteosarcoma. Thus, the current study aims to determine the proportion of expression of *RANKL* in osteosarcoma and its association with the clinicopathological data. 

## Materials and Methods

All patients diagnosed histologically as osteosarcoma from the year 2003 to 2018 in Hospital Universiti Sains Malaysia (Hospital USM) were included in the study using a convenience sampling method. The cases were retrieved via computerized record collection system (PATHORS) and Laboratory Information System (LIS) from the Department of Pathology, Hospital USM. The clinicopathological data were retrieved from medical records, histopathology reports, and computerised radiology imaging (PACS).

Tumour volumes were calculated using three parameters (length, width, and depth) by using the ellipsoid formula [V=(π/6)abc] (Munajat et al., 2008). For staging, the classification developed by Enneking was utilised (Faisham et al., 2017). The specimen’s pathologic subtypes were based on WHO classification of Tumours of Soft Tissue and Bone (Fletcher et al., 2013). The response to chemotherapy was assessed microscopically. Patients who were good responders have equal to or greater than 90% tumour necrosis, while those with less than 90% tumour necrosis based on histopathological reports were considered poor responders (Faisham et al., 2017). Recurrence was defined as osteosarcoma diagnosed histopathologically from a similar site of the initial tumour. Disease-free survival (DFS) was defined as the time between primary surgery and the first evidence of tumour progression. Osteosarcoma-specific survival (OSS) was defined as the time between the patient’s primary surgery and death due to tumour progression (Bago-Horvath et al., 2014). The patients’ clinicopathological data were recorded in a separate proforma and kept in Statistical Package for the Social Sciences (SPSS) version 24.

The selected formalin-fixed paraffin-embedded tissue blocks were sectioned and stained with both Hematoxylin and Eosin (HandE) and RANKL antibody according to standard procedures (Abcam Code ab9957). For RANKL staining, the tissue slides were heated on the hotplate at 60°C for 1 hour and proceeded for hydration process and antigen retrieval process in PT link (Dako). The endogenous peroxidase was blocked by incubation in 3% hydrogen peroxidase and protein block (Abcam Code ab64226) for 10 minutes each at room temperature. Rabbit polyclonal primary antibody to RANKL with dilution 1:250 was applied to the sections and incubated overnight at room temperature. Chromogen was applied to the sections and incubated for 5 minutes, followed by counterstaining with hematoxylin. Finally, the slides were mounted with cytoseal. Sections without the primary antibody staining served as negative control while lymph node tissue was used as a positive control. The slides were viewed using a standard light microscope Olympus CX-31. 

*RANKL* expression in the cytoplasm and cell membrane of the tumour cells were examined using a scoring method (Bago-Horvath et al., 2014). A score combining the percentage of positively stained cytoplasm and cell membrane of the tumour cells (0, none; 1, 1–29%; 2, 30–69%; 3, 70–100%) with their intensity (0, none; 1, weak; 2, moderate; 3, strong) was constructed. A three-tiered system (negative, weakly positive, strongly positive) was applied to evaluate the staining results, as shown in Table 1. Tumours were considered positive if the sum of scores was >2. Weakly positive cases were defined by a sum of scores 3–5. A sum of scores >5 defined strongly positive cases. Staining results were independently evaluated by one pathologist blinded to the patient’s clinicopathological status. Kaplan-Meier test was used to describe the median survival time of the patients. Chi-Square and Fisher Exact tests were done to identify the association of immunopositivity of RANKL with the clinicopathological parameters. The level of significance in this study was set as p-value <0.05. 

## Results

The clinicopathological characteristics of the total 100 patients are shown in Table 2. The age ranges from 2 to 70-year-old with the mean age of the patients were 20.4 years old. The commonest age is 15 years old. Eighty-six percent of them were Malay ethnicity, male patients (62%) are more common than females. There were 66.2% (49/74) of patients presented with bone swelling, while 33.8% (25/74) presented with bone pain. The femur is the commonest site of presentation, 46.4% (45/97), followed by the tibia, humerus, and other locations. Most of the cases were diagnosed at stage IIB, which accounted for 67.1% (51/76) by Enneking staging system. The majority (92%) were conventional osteosarcoma, and mostly (98%) were high grade. 

A total of 82 cases were positive for *RANKL *expression immunohistochemically, with 56 and 26 of them were weakly and strongly positive, respectively. Eighteen percent (18%) of the cases recorded negative *RANKL *expression. However, most clinicopathological characteristics did not show a statistically significant association with the *RANKL* expression (Table 3). 

**Figure 1:**
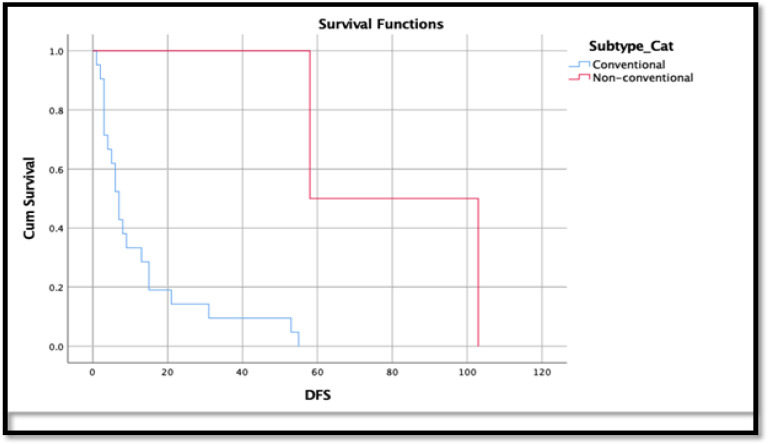
Cumulative Disease-Free Survival (DFS) in Conventional vs. Non-Conventional Osteosarcoma with p-value 0.01

**Figure 2:**
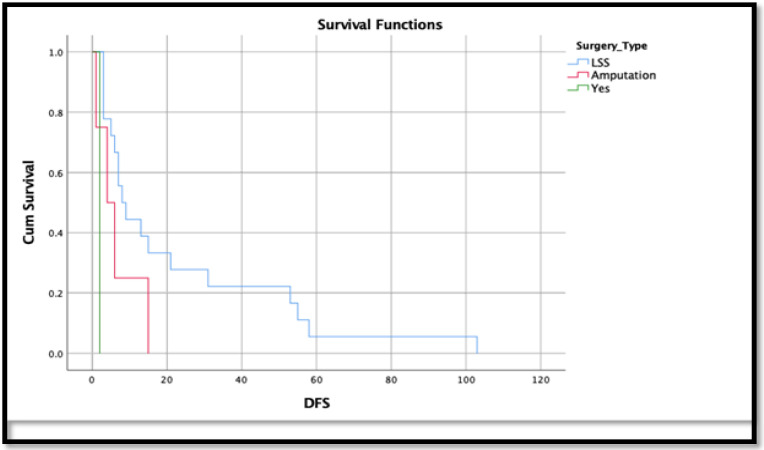
Cumulative Osteosarcoma-Specific Survival (OSS) (n=13) in Patients who were Amputated vs. Underwent Limb Salvage Surgery (LSS). p-value 0.002

For median DFS and OSS calculation, only 23 patients and 13 patients were included, respectively due to incomplete medical records. The median DFS for 23 patients who ended with tumour progression was 13 months (RANKL negative) and seven months (RANKL positive) (Figure 1). The median OSS for 13 patients who resulted in death was 11 months (RANKL negative) and 19 months (RANKL positive). However, Kaplan Meier survival analysis showed a significant improvement in the disease-free survival patients who underwent limb salvage surgery (LSS) than amputated patients (p value 0.002), whereas poorer survival was observed among conventional osteosarcomas compared to non-conventional osteosarcoma (p-value 0.01) (Figures 1 and 2). 

**Table 1:** Scoring System for RANKL Expression

Score	Percentage of positive tumour cells (A)	Intensity of stain on cytoplasm and cell membranes (B)
0	0	None
1	1–29%	Weak
2	30–69%	Moderate
3	70–100%	Strong

Score = A + B; Positive expression if the sum of scores was >2; Weakly positive cases were defined by a sum of scores 3–5; Strongly positive cases were defined by a sum of scores >5.

**Table 2:** Clinicopathological Characteristics (n=100).

Variables	Mean (SD)		n (%)		
Clinicopathological characteristics					
Age (year)	20.4 (11.76)				
Age group (year)					
< 40			92 (92)		
≥ 40			8 (8)		
Gender					
Male			62 (62)		
Female			38 (38)		
Race					
Malay			86 (86)		
Chinese			9 (9)		
Indian			5 (5)		
Family history with osteosarcoma (n=30)*					
No			29 (96.7)		
Yes			1 (3.3)		
Presentation (n=74)*					
Bone pain			25 (33.8)		
Swelling			49 (66.2)		
Tumour volume (n=75)*					
< 150 ml			23 (30.7)		
≥ 150 ml			52 (69.3)		
Tumour site (n=97)*					
Femur			45 (46.4)		
Tibia			28 (28.9)		
Humerus			7 (7.2)		
Maxillofacial			2 (2.1)		
Pelvic			5 (5.2)		
Others			10 (10.3)		
Stage of disease (Enneking) (n=76)*					
IA			-		
IB			-		
IIA			2 (2.6)		
IIB			51 (67.1)		
III			23 (30.3)		
Pathologic subtypes					
Conventional			92 (92)		
Low-grade central			1 (1)		
Telangiectatic			5 (5)		
Small cell			1 (1)		
Parosteal			1 (1)		
Grade					
Low			2 (2)		
High			98 (98)		
Metastatic event (n=94)*					
No			38 (40.4)		
Yes			56 (59.6)		
Surgery (n=88)*					
No			15 (17)		
Yes			73 (83)		
Variables	Mean (SD)		n (%)		
Chemotherapy (n=89)*					
No			24 (27)		
Yes			65 (73)		
Response to chemotherapy (n=43)*				
Non-Responder				31 (72.1)
Responder				12 (27.9)
Recurrence (n=96)*				
No				86 (89.6)
Yes				10 (10.4)
Disease-free-survival in months (n=23)*		18.7 (25.36)		
Osteosarcoma-specific- survival in months (n=13)*		17.6 (12.03)		

*Not all the patients had complete clinicopathological data

**Table 3:** Association between RANKL ImmunohistoChemistry Expression in Osteosarcoma with the Clinicopathological Characteristics Using Pearson Chi-Square (n=100).

Variables	RANKL negative n (%)	RANKL positive n (%)	p value
Clinicopathological characteristics			
Age group (year)			
< 40	16 (17.4)	76 (82.6)	0.632†
≥ 40	2 (25)	6 (75)	
Gender			0.774
Male	13 (21)	49 (79)	
Female	5 (13.2)	33 (86.8)	
Family history with osteosarcoma (n=30)*			0.48†
No	5 (17.2)	24 (82.8)	
Yes	0 (0)	1 (100)	
Presentation (n=74)*			0.342†
Bone pain	6 (24)	19 (76)	
Swelling	7 (14.3)	42 (85.7)	
Tumour volume (n=75)*			0.96†
< 150 ml	5 (21.7)	18 (78.3)	
≥ 150 ml	9 (17.3)	43 (82.7)	
Tumour site (n=97)*			
Femur	9 (20)	36 (80)	0.143†
Tibia	3 (10.7)	25 (89.3)	
Humerus	0 (0)	7 (100)	
Maxillofacial	0 (0)	2 (100)	
Pelvic	3 (60)	2 (40)	
Others	2 (20)	8 (80)	
Stage of disease (Enneking) (n=76)*			
IA	-	-	0.18†
IB	-	-	
IIA	0 (0)	2 (100)	
IIB	11 (21.6)	40 (78.4)	
III	4 (17.4)	19 (82.6)	
Grade			
Low	1 (50)	1 (50)	0.329†
High	17 (17.3)	81 (82.7)	
Metastatic event (n=94)*			0.36
No	7 (18.4)	31 (81.6)	
Yes	9 (16.1)	47 (83.9)	
Surgery (n=88)*			0.064†
No	0 (0)	15 (100)	
Yes	15 (20.5)	58 (79.5)	
Chemotherapy (n=89)*			1.000†
No	4 (16.7)	20 (83.3)	
Yes	12 (18.5)	53 (81.5)	
Response to chemotherapy (n=43*)			0.69†
Non-Responder	10 (32.3)	21 (67.7)	
Responder	1 (8.3)	11 (91.7)	
Recurrence (n=96)*			1.000†
No	15 (17.4)	71 (82.6)	
Yes	1 (10)	9 (90)	

*, Not all the patients had complete clinicopathological data; †, Fisher's Exact Test.

**Figure 3:**
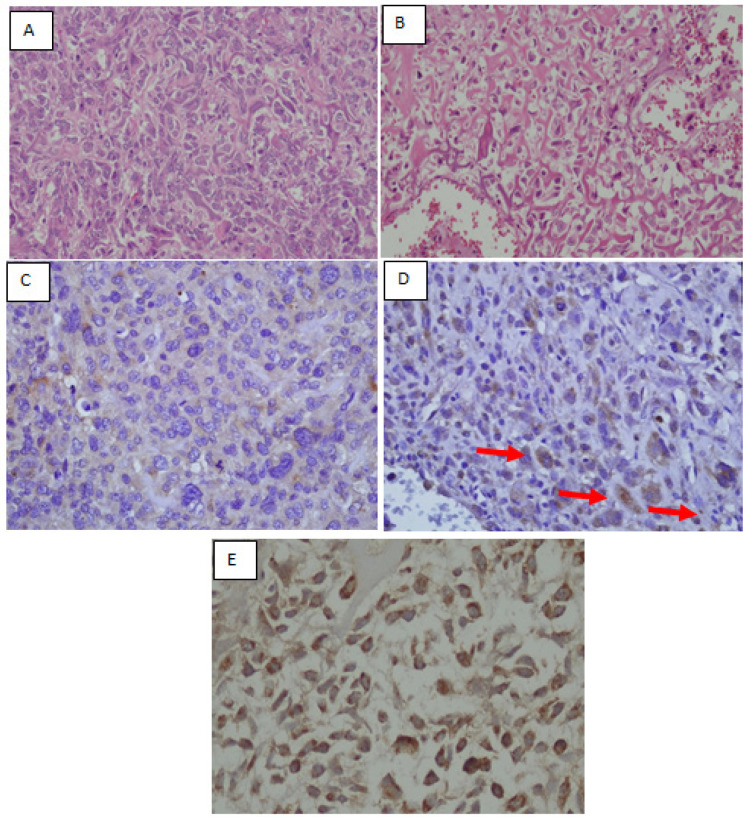
(A) H&Ex200; The osteosarcoma cells exhibited cell pleomorphism with (B) H&Ex200; lace-like osteoid formation. (C) Osteosarcoma cells with RANKL negative, (D) low expression of RANKL, and (E) high expression of RANKL (IHC x400). The positivity of RANKL refers to cytoplasmic and membrane immunoreactivity by IHC (red arrows).

## Discussion

The present study reports the proportion of *RANKL* expression among osteosarcoma specimens and evaluates the association between *RANKL* expression and the patients’ clinicopathological characteristics. *RANKL* expression in other bone tumours has been studied to determine its role as a reliable prognostic marker in predicting recurrence (Ghani et al., 2018) and the use of Denosumab in treating patients with *RANKL* expression (Heymann, 2012). However, there were conflicting reports on the *RANKL* expression in osteosarcoma in a few limited studies. 

Our study reported up to 82% of cases with positive expression of *RANKL* with 56% and 26% of them were weakly and strongly positive, respectively. This is relatively high compared to other similar studies on osteosarcoma from other parts of the world, with a reported proportion from 8.8% to 75% (Lee et al., 2011; Bago-Horvath et al., 2014; Branstetter et al., 2015). Bago-Horvath et al., (2014) used the same method of reporting positivity of *RANKL* expression, but they only reported 8.8%. The difference might be due to the different antibody clones used as they were using R and D Systems, USA; 1:100. Difficulty in antibody optimization such as high background staining was noted in our cases, which initially was thought to be caused by non-specific binding. Repeated staining was done with a longer incubation period of peroxidase blocking agent, but the result was similar. Thus, we have changed the anti-RANKL antibody from monoclonal to polyclonal antibody with added protein blocking process and increased the blocking incubation period at room temperature together with the dilution of primary antibody concentration done accordingly. Therefore, the confirmation of RANKL status should be verified by a more sensitive and specific molecular method. 

Studies showed that healthy older people aged between 81 and 90 years have a lower level of soluble RANKL and people living with osteoarthritis and polymyalgia rheumatica (Pulsatelli et al., 2004). Also, Kerschan-Schindl et al., (2008) showed that the serum RANKL levels decrease by approximately 13% by every five years. Our study illustrated the findings on tissue sample also support this finding as only six patients with RANKL positive were 40 years old and above, compared to 76 patients aged younger than 40 who had a positive expression of *RANKL* is not statistically significant.

Osteosarcoma most frequently occurs in people in the second decade of life; about 60% of patients were below 25. But 30% of osteosarcomas did occur in patients over 40 years of age (Fletcher et al., 2002). We studied 100 patients in our center, only 8% of patients were diagnosed after 40 years old. The commonest age is 15 years old (10%). 

Male patients are more frequently affected than female patients in a ratio of 3:2 for conventional osteosarcoma, especially for those under 20 years of age (Fletcher et al., 2002), which is comparable with our study with 62 male patients and 38 female patients. Meanwhile, Kerschan-Schindl et al., (2008) measured the serum level of RANKL in healthy people who have no chronic diseases or using any medication that can affect bone metabolism showed that men have a 1.77-fold higher free RANKL level on average. In our study for *RANKL* expression on tissue, those with positive *RANKL* expression, 49 were men while 33 were women at a lower ratio of 1.48. Male patients have a higher risk indicating that hormonal changes, bone growth, and puberty development may be involved in osteosarcoma’s pathophysiology (Savage and Mirabello, 2011).

There were 66.2% (49/74) of patients presented with bone swelling, while 33.8% (25/74) presented with bone pain. This finding was different from other studies, whereby pain was the main presentation (Younger et al., 2018). It may be due to the pain was insidious and intermittent at the initial presentation, and the patients only seek treatment when swelling appeared.

There were 46.4% (45/97) of patients who had osteosarcoma of the femur which was the most common site of osteosarcoma and 67.1% (51/76) of them were diagnosed at stage IIB using Enneking staging system. These findings are consistent with the results from the previous study, which showed femur as the primary site that accounts for 43% and 39% of their cases were diagnosed at stage IIB which was highest (Branstetter et al., 2015). 

Consistent with Lee et al., (2011), we showed that most cases with *RANKL* expression have a poorer response to chemotherapy, which can be used as an important prognostic factor. However, our result did not show any significant association for *RANKL* expression with chemotherapy response. 

Since the current study showed that RANKL is highly expressed in osteosarcoma tissue, this technique may be effective in routine staining of new cases with osteosarcoma in the future. There was a case report using Denosumab as RANKL inhibitor and tyrosine kinase inhibitor Sorafenib to treat a case of chemo-refractory osteosarcoma with an overexpression of *RANK* and RANKL, showing they were able to achieve a complete metabolic remission (Cathomas et al., 2015). A recent study, Chen et al., (2015), reported that RANKL blockade was effective for the management of osteosarcoma in animal models.

The oncological and functional outcomes of limb salvage surgery (LSS) combined with chemotherapy are the better option for osteosarcoma patients. Our data have shown a significant improvement in disease-free survival of patients who underwent LSS than amputation, supported by (Tiwari, 2014). In the present study, a significant number of patients with osteosarcoma have a positive expression for *RANKL*. However, our data did not show a statistically significant association with any of the clinicopathological characteristics and patients’ final survival. Further studies may be acquired to understand the suitability of using RANKL immunohistochemistry as prognostication in managing osteosarcoma patients. Furthermore, the role of denosumab and bisphosphonate as an adjuvant treatment of osteosarcoma has questionable benefits.

## Author Contribution Statement

The manuscript has been read and approved by all the authors. All authors were involved in data analysis and manuscript draft preparation. 
